# A Special Section of Articles From the 8^th^ Annual Bio-Ontologies Meeting

**DOI:** 10.1002/cfg.502

**Published:** 2005


					Comparative and Functional Genomics
A Special Section of articles from
The 8th
Annual Bio-Ontologies Meeting
A Special Interest Group (SIG) Workshop of the
13th Intelligent Systems for Molecular Biology (ISMB)
Held at the
Renaissance Center Marriott, Detriot, Michigan, USA
24th June 2005
Edited by
Robert Stevens and Phillip Lord
Workshop Organisers
Robert Stevens (School of Computer Science, University of Manchester, UK)
Robin McEntire (GlaxoSmithKline, King of Prussia, USA)
Phillip Lord (School of Computer Science, University of Manchester, UK)
James.A.Butler (GlaxoSmithKline, King of Prussia, USA)

Current address: School of Computing Science, University of Newcastle, UK
The Bio-Ontologies workshop has been a satellite meeting to the annual ISMB conference
since 1998, and is now operated as a Special Interest Group at the ISMB Conference. Bio-
Ontologies is well established as one of the key meetings for dissemination of latest information
and research on ontologies in the life sciences and regularly draws many researchers in the
field.

				

